# The Effects of Feeding Waste Milk Containing Antimicrobial Residues on Dairy Calf Health

**DOI:** 10.3390/pathogens10020112

**Published:** 2021-01-22

**Authors:** Clair L. Firth, Katrin Kremer, Thomas Werner, Annemarie Käsbohrer

**Affiliations:** Unit of Veterinary Public Health and Epidemiology, Institute of Food Safety, Food Technology & Veterinary Public Health, University of Veterinary Medicine, 1210 Vienna, Austria

**Keywords:** cattle, antimicrobial resistance, mastitis, dairy, *Escherichia coli*, veterinary public health, waste milk, antibiotics

## Abstract

A number of studies have reported that there is a high prevalence of antimicrobial-resistant faecal bacteria excreted by dairy calves. Although faecal shedding is influenced by a variety of factors, such as the environment and calf age, feeding milk with antimicrobial residues contributes significantly to an increased prevalence of antimicrobial-resistant (AMR) bacteria, such as extended spectrum beta-lactamase (ESBL)-producing *E. coli*. As a follow-up to the European Food Safety Authority (EFSA) Scientific Opinion on the risk of AMR development in dairy calves published in January 2017, this review aims to illustrate more recent research in this area, focusing on the period 2016 to 2020. A total of 19 papers are reviewed here. The vast majority assess the commensal faecal bacteria, *E. coli*, isolated from dairy calves, in particular its antimicrobial-resistant forms such as ESBL-producing *E. coli* and AmpC-producing *E. coli*. The effect of waste milk feeding on the prevalence of pathogens such as *Salmonella* spp. has also been investigated. Current research findings include positive effects on daily liveweight gain and other advantages for calf health from feeding waste milk compared to milk replacer. However, the negative effects, such as the demonstrable selection for antimicrobial-resistant bacteria, the shift in the intestinal microbiome and the possible negative consequences that these could have on global public health, should always be taken into consideration.

## 1. Introduction

The use of antimicrobials and the subsequent selection for antimicrobial resistance in commensal and pathogenic bacteria is a global health problem affecting both human and veterinary medicine as well as the wider human and animal populations and the environment [1,2].

On a global scale, dairy cows are frequently treated with antimicrobials, with mastitis being the most common indication for the use of such veterinary medications [3,4,5,6]. Whenever dairy cows are treated with medicinal products that are licensed with a withdrawal period (during which time the milk cannot be delivered to the commercial dairy for human consumption), waste or discard milk is produced. This waste milk is sometimes dumped into watercourses, drains or onto manure heaps but is more commonly fed to calves [3]. The continuous feeding of small amounts of antimicrobial residues in waste milk is a potential cause for concern with respect to the development of antimicrobial resistance, and for this reason, in 2017, the European Food Safety Agency (EFSA) published a “Scientific Opinion” report on the possible risks posed by such feeding [3]. As part of this report, the European Commission sent a questionnaire to representatives of all EU member states and asked them to describe the legal situation regarding waste milk feeding in their respective countries. Almost all (27) of the 28 EU nations reported no specific legislation or chose to provide no information on this subject; only Austria reported that the feeding of waste milk was restricted by law to milk from the calf’s own dam [3]. Very few countries had any official data on the level of waste milk feeding. The EFSA experts estimated that approximately 1% of the milk produced in the European Union can be classed as non-saleable (“waste milk”) and is likely to be fed to calves [3]. However, the overall conclusions of the ESFA report were that while feeding calves with colostrum containing certain antimicrobial residues did not increase faecal shedding of AMR bacteria, the feeding of waste milk to calves has a transient effect on the faecal shedding of AMR bacteria, which reduces over time, and measures should be taken to inactivate antimicrobial residues and/or AMR bacteria prior to feeding [3]. As the EFSA Scientific Opinion was published in January 2017, the aim of this narrative literature review is to provide an updated overview of the scientific research in this area published since 2016.

## 2. Results

### 2.1. Narrative Literature Review

The results of the narrative literature review are presented here. Additional adjustments to the search log are noted below. Although this was not a systematic review, the flowcharts shown are based on the PRISMA template [7]. As a narrative literature review, the results included here aimed to provide an overview of the effects of waste milk feeding on the selection of antimicrobial-resistant bacteria and the overall influence on calf health. Unlike a systematic review, this review did not focus solely on answering one specific research question but aimed to provide readers with a synopsis of the current scientific literature. For this reason, some research may have been overlooked by the use of specific keywords, and the potential for inclusion bias must be considered.

**Figure 1:**
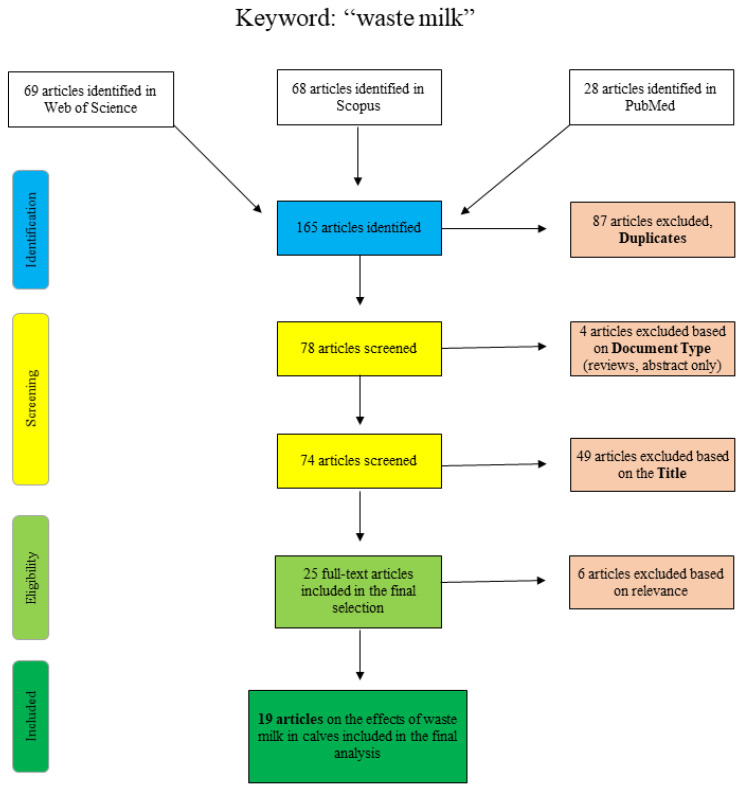
Flowchart for the search term “waste milk”.

Figure 1 shows the articles found and sorted according to specific criteria for the search term “waste milk”. The articles of PubMed were collected by “title/abstract: waste milk” and the additional restriction “Species: other animals”, so that articles concerning human lactation were excluded. The Scopus database was scanned as follows: “title/abstract/keywords: waste-milk”, so that the term only appears in a coherent way as well as “title/abstract/keywords: waste AND milk AND antibiotic” and “title/abstract/keywords: waste AND milk AND calf”. Web of Science articles were identified using the terms “waste AND milk” or “waste milk” as well as different combinations of the terms “calf”, “antimicrobial” and/or “antibiotic”. Articles that were excluded on the basis of the title clearly did not deal with the topic of waste milk in a veterinary or relevant agricultural context (e.g., waste management (recycling), food chemistry or soil science).

**Figure 2:**
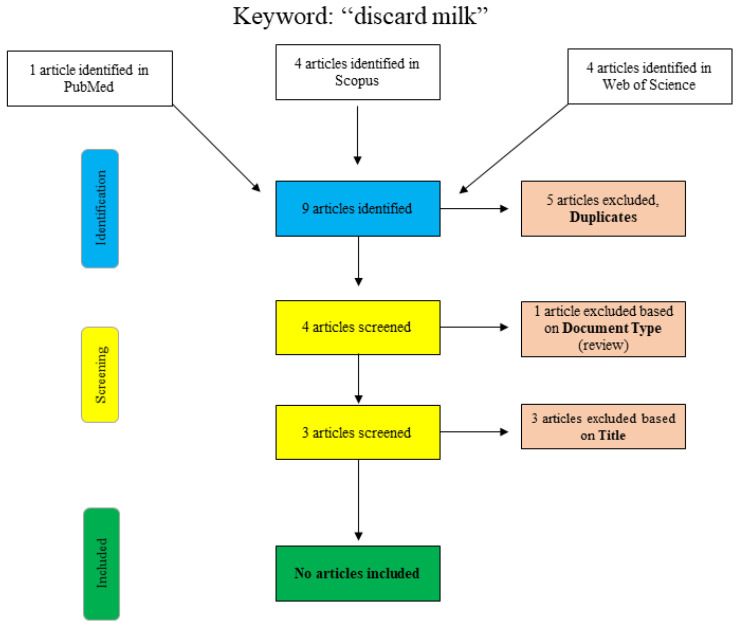
Flowchart for the search term “discard milk”.

A further search for the term “discard milk” is shown graphically in Figure 2. In PubMed, the search was performed using the search term “discard milk” and the limitation to “species: other animals”. The articles in Scopus and Web of Science were found using “title/abstract/keywords: ‘discard milk’”. The three final articles that were excluded with respect to the title were clearly not relevant to the topic of this narrative review, and no additional articles were included. 

### 2.2. Summary of the Findings of the European Food Safety Agency Scientific Opinion Entitled “Risk for the Development of Antimicrobial Resistance (AMR) Due to Feeding of Calves with Milk Containing Residues of Antibiotics”

The risk assessment included in the EFSA Scientific Opinion is based on the excretion of resistant bacteria in calf faeces [3]. The results of the studies available prior to 2016 are consistent with regard to an increased excretion of resistant bacteria when feeding waste milk [8,9,10,11,12,13]. Thames and colleagues [14] were the only researchers included in the EFSA Scientific Opinion who could not demonstrate a significant difference in the proportion of resistance genes present dependent on waste milk feeding regime compared to unmedicated milk replacer. However, unlike the other studies, Thames used a non-culture method, namely, quantitative PCR, for certain AMR genes rather than analysing phenotypic AMR. 

The feeding of colostrum from cows treated with antimicrobial dry cow therapy was also investigated by EFSA. The studies by Swedish (Duse and colleagues) and Dutch researchers (Gonggrijp and colleagues—study not published but provided to EFSA experts) mentioned in the expert opinion demonstrated that there was no link between this type of colostrum feeding and an increased excretion of resistant *E. coli* in calves [3,10].

Regarding the duration of excretion of AMR bacteria by dairy calves, a significant increase in the excretion of resistant bacteria was described at two to three weeks of age [8,12,13]. Several studies subsequently showed that initial shedding of such bacteria reduced over time, e.g., by seven weeks of age in Germany [8] and by twelve weeks (six weeks after weaning) in the UK, leading to no difference between calves originally fed waste milk and the control group [12]. A further Swedish study by Duse and colleagues demonstrated a lower level of AMR bacteria in older calves regardless of previous feed regimen [10]. In contrast, a study by Berge and colleagues in the USA concluded that there is increased resistance formation with increasing age, independent of feeding regime [9].

The EFSA experts point out that the increased excretion may also be influenced by other factors, such as the environment [3]. A number of authors also reported that adult cows were much less likely to shed AMR bacteria than young calves [15,16]. Furthermore, Swedish researchers led by Duse showed that selection for resistance was independent of whether the milk fed was collected during treatment with antibiotics or during the subsequent withhold period [10]. Pasteurisation of waste milk fed to calves was reported not to influence the excretion of resistant bacteria compared to untreated waste milk [8].

The EFSA experts also investigated to what extent feeding either waste milk or commercial milk/milk replacer has influenced weight gain, feed intake and health parameters. No effect could be determined with respect to weight gain [8,11,14], calf health parameters [8,14] or feed intake [8].

The direct effect of waste milk feeding on the calf’s microbiome is also discussed. Studies by Lalles, Yeoman and White, and Mao and colleagues have shown that a reduction in the diversity of intestinal microbes leads to poorer health, which also has far-reaching consequences for the development of the immune system [17,18,19].

### 2.3. Literature Published on Antimicrobial Resistance and Waste-Milk Feeding in Dairy Calves from 2016–2020

**Figure 3:**
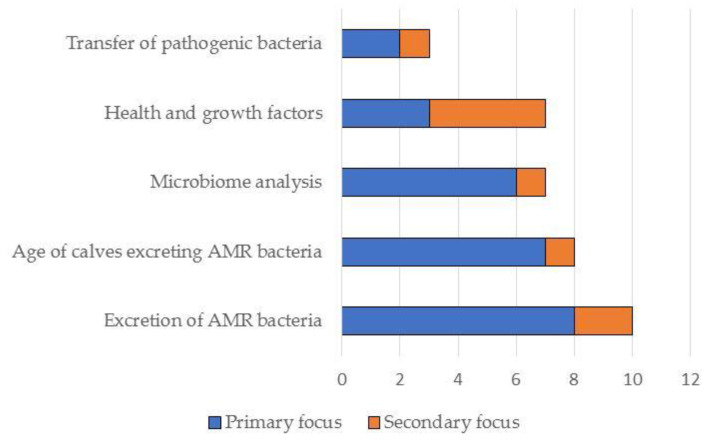
Summary of the primary and secondary focus of the studies included in this narrative review (*N* = 19).

**Figure 4:**
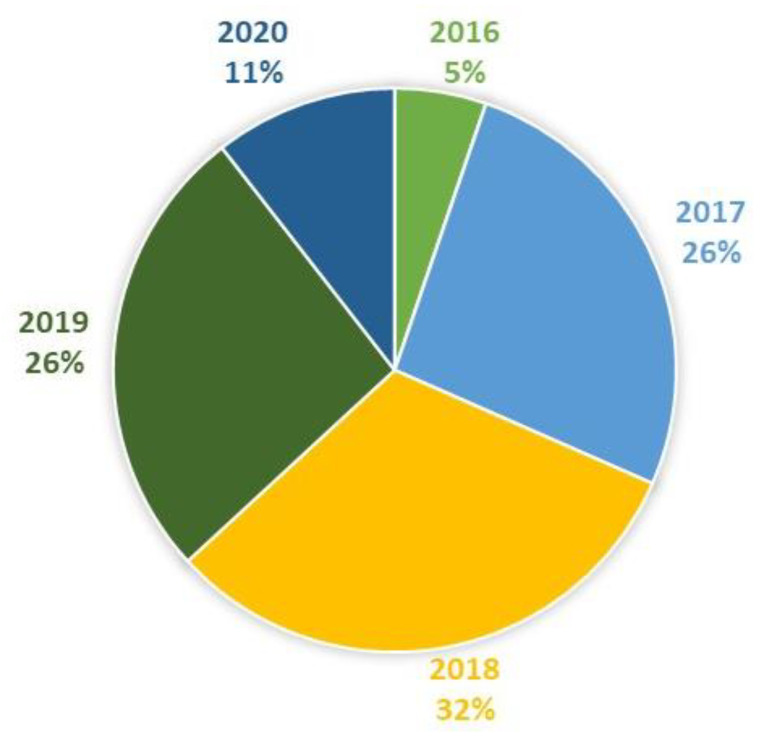
Studies included in this review by year (*N* = 19).

**Figure 5:**
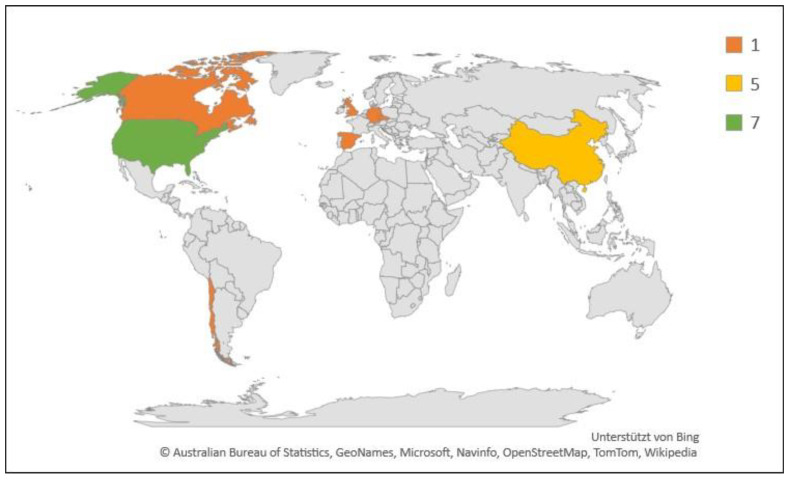
Geographic and numerical distribution of studies included in this review (*N* = 18). Legend indicates number of studies.

**Figure 6:**
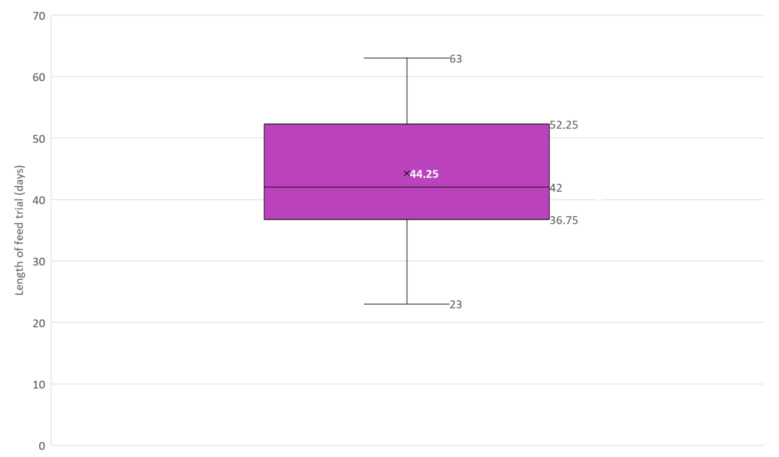
Box and whisker plot of the length in days of feed trials included in this narrative review (*N* = 12). × = mean, horizontal line = median (Q2), box = the range between the 1st (Q1) and 3rd (Q3) quartile; lower whisker, Q1 − 1.5 (IQR) (interquartile range); upper whisker, Q3 + 1.5 (IQR).

In total, the literature search resulted in the identification of 25 studies on the effects of feeding waste milk to calves. Of these, 19 were relevant to this narrative review and are discussed in detail below (see also Figure 3, Figure 4, Figure 5 and Figure 6). Further details on study design, sample size calculations, control groups, etc., are shown in Table 1 and Tables S1 and S2 in the Supplementary Materials.

## 3. Discussion

A total of 19 relevant studies have been published since 2016 on the subject of waste/discard milk, antimicrobials and calves and are included in this narrative literature review. 

It is important to note that studies on the effects of waste milk feeding in dairy calves are often hampered by the need to be practically relevant to farmers balanced with the requirement to obtain scientifically valid results. Waste milk, also known as ”non-saleable” or discard milk, may be colostrum, obtained from transition cows shortly after calving, or from lactating cows treated either systemically or locally (intramammary) with antimicrobials [3]. Waste milk may also refer to milk from cows treated with other, non-antimicrobial, drugs such as NSAIDs for the treatment of pain and inflammation. For these reasons, surveys that ask producers about waste milk feeding of calves may actually be referring to a variety of milk types. While pasteurisation may be used to reduce the level of potentially pathogenic bacteria in waste milk, heat treatment is often not adequate to remove antimicrobial residues from this milk source [39]. Some of the studies included in this narrative review have, therefore, used experimentally produced waste milk that has been “spiked” with antimicrobials, while others use naturally produced waste milk from cows treated with antimicrobials. In addition to variations in the level of antimicrobial residues, it should be noted that natural waste milk may also vary in milk composition from cow to cow and farm to farm. 

### 3.1. Excretion of Resistant Bacteria (Both Commensal Bacteria and Pathogens)

A longitudinal study by Horton and colleagues in the United Kingdom investigated the presence of extended spectrum beta-lactamase (ESBL)-producing *E. coli* on a single farm with 250 Holstein–Friesian cows and 40 unweaned calves [27]. Fresh faecal samples were collected from 25 calves every two weeks for a total of six visits; samples were not collected rectally but from fresh pats on the barn floor. All calves were fed with waste milk from either mastitic cows or freshly calved cows that had previously received antimicrobial dry cow therapy (DCT). Presumptive CTX-M ESBL-producing *E. coli* were detected at extremely high frequencies in calf samples at all visits (ranging from 80–100%, overall 93%) [27]. Samples taken from the calving pen approximately one year later were also 100% positive for this AMR pathogen. Three samples of waste milk tested one year later were found to contain residues of cefquinome and cephalexin; two out of the three waste milk samples were also positive for presumptive CTX-M ESBL-producing *E. coli* [27]. Ten calves in this study were individually assessed for excretion of presumptive CTX-M ESBL-producing *E. coli* over time and were found to shed these AMR pathogens for a maximum of 64 days (median 36 days), excretion frequency fell once calves were weaned [27].

A study by Manga and colleagues in the Czech Republic analysed the faecal excretion in dairy calves of cephalosporin-resistant *E. coli* pathogen [29]. A single farm with 620 Holstein cows was included in this field study. Antimicrobial usage on the farm in defined daily doses (DDD) per cow and year was collected by interviewing the herd veterinarian and farm manager and was reported to be highest for lincomycin, with the highest priority critically important antimicrobials (HPCIAs) marbofloxacin, cefquinome and cefoperazone used at a lower levels [29]. Over a two-month period, faecal samples from 13 clinically healthy calves were repeatedly tested for the presence of cephalosporin-resistant *E. coli*. Using selective media, cephalosporin-resistant *E. coli* were found in faecal samples from all 13 calves at all sampling periods (including 1–2 days post-partum). Two of the dams also tested positive for this resistant type of *E. coli*. Most (82/87; 94%) of these cefotaxime-resistant *E. coli* were found to produce AmpC. Whole genome sequencing of 10 selected isolates showed the presence of a bla_CMY-2_ gene confirming AmpC production. No ESBL-producing *E. coli* were isolated. The calves all received unpasteurised colostrum after birth and were then fed milk or pasteurised colostrum twice a day. It is important to note that the study authors do not explicitly state that waste milk was fed, but they do mention that milk contained “antibiotic residues”. The farm used selective dry cow therapy and none of the calves included in the study had been treated with antimicrobials for infection. No cefotaxime-resistant bacteria were isolated from milk samples collected, although the initial feed of unpasteurised colostrum was not tested and may have been a source of colonisation. Unlike some other studies of this type (e.g., Maynou and colleagues [31]), the authors reported that the faecal shedding of cefotaxime-resistant *E. coli* did not appear to reduce over time, being present up to 63 days post-partum [29].

An increased level of excretion of resistant bacteria in the first weeks of a dairy calf’s life, as well as a subsequent reduction in this effect, was also reported in the studies included in the EFSA Scientific Opinion [8,12,13,15,16,40]. In contrast, Thames and colleagues could not confirm this effect, but the calves were only sampled from six weeks of age [14]. Berge and colleagues also described an increase in the excretion of resistant bacteria within the first three weeks of life; however, no further samples were taken at a later stage [9]. 

Researchers from Spain (Maynou and colleagues) investigated the resistance development of *E. coli* in faeces and *Pasteurella multocida* (*P. multocida*) in nasal samples of calves when fed (unmedicated) milk replacer in comparison to waste milk [30]. Eight commercial farms fed either waste milk (containing either antimicrobial residues or high somatic cell count (SCC), in the case of mastitis milk) or milk replacer (four farms per feed group). Calves that received antimicrobial treatment for disease were excluded. Samples were taken from 20 ± 5 calves after six weeks and again from around 10 calves at one year of age. In the faeces of the calves that received waste milk, an increased occurrence of resistant and multi-resistant *E. coli* was detected. Statistically significant higher resistance levels were detected in calves from the waste milk feeding group at six weeks to enrofloxacin (*p* < 0.01), florfenicol (*p* < 0.05) and streptomycin (*p* < 0.05). The nasal swab samples showed a higher proportion of resistant *P. multocida* to colistin in the group fed with waste milk. Overall, this study showed that the feeding of waste milk under these conditions led to an increased prevalence of antimicrobial-resistant bacteria [30]. The study also demonstrated the transient nature of such AMR bacteria shedding. An increasing prevalence of resistance to enrofloxacin and doxycycline up to the sixth week of age could be demonstrated independent of the feeding regime, but in the case of doxycycline, this decreased again from the sixth week of age onwards. Resistance to enrofloxacin and streptomycin was demonstrably higher in the sixth week (compared to one year later) when waste milk was fed, as was the prevalence of multi-resistant *E. coli* [30].

This age-related decrease in the excretion of resistant bacteria was also confirmed in a separate study by the same Spanish researchers when waste milk was fed [31]. Here, the excretion of *E. coli* when feeding pasteurised waste milk containing β-lactam residues was investigated in comparison to milk replacer without antimicrobial residues. Again, calves receiving antimicrobial treatment (prior to 42 days of age) were excluded from the study. The change in the resistance pattern before and after weaning on day 49 was compared by taking faecal samples on days 0, 35 and 56. The presence of resistance to 12 classes of antimicrobials was tested by the disc diffusion method, and the presence of 13 different resistance genes was investigated by PCR. Isolates from faecal samples from both groups were found to be resistant to tetracyclines, sulfonamides and aminoglycosides, regardless of feed management, suggesting other factors also play a role in the occurrence of antimicrobial resistance. The resistance to β-lactams was, however, comparatively higher in the group fed with waste milk. Furthermore, calves fed milk containing β-lactam residues were more likely to have florfenicol-resistant *E. coli* in their faeces than those fed milk replacer [31]. This longitudinal study also demonstrated that the presence of AMR-*E. coli* decreases with calf age [31]. The investigated resistance genes to β-lactams (ampicillin, ceftiofur, cephalothin) were detected one day post-partum with a prevalence of 12.5%. By the 35th day of age, only 2.2% of calves were excreting these resistant bacteria in their faeces. 

A Canadian study led by Awosile investigated the presence of *Salmonella enterica* and extended spectrum cephalosporin-resistant (ESC-R) *E. coli* in the faeces of 488 dairy calves on eight farms [20]. Faecal samples were collected as rectal swabs from the calves at 2–15 and 42–56 days of age and cultured selectively, followed by antimicrobial susceptibility testing and PCR analysis to assess the β-lactamase resistance genes further. Data on farm management (such as the feeding of waste milk) were collected by questionnaire. Only 3.3% of the 488 calves tested were shedding *Salmonella enterica* (i.e., had a positive result on at least one of the two test periods), and these calves were significantly more likely to be neonates than of weaning age (*p* < 0.05) [20]. The low level of *Salmonella* spp. isolated precluded further statistical tests and led the authors to believe that this pathogen was not of particularly high risk for cattle in this region. Based on selective cultures, ESC-R *E. coli* were isolated from 81.2% of calves, with just over half of these calves being positive at both the neonatal and weaning period sampling. Multidrug resistance (i.e., resistance to at least one antimicrobial in at least > 3 antimicrobial groups) was determined in 88.0% of selected ESC-R *E. coli* tested [20]. More recent whole-genome sequence analysis of bla_CMY2_ *E. coli* isolates obtained from four calves in this Canadian study by Awosile and colleagues has demonstrated that three of four isolates represent ST88 (cloned complex 23), which is known to transfer between species (human and animal), all four isolates also carried a variety of genes for antimicrobial resistance, namely, bla_CMY-2_, bla_TEM-1B_, tetA, tetB, tetD, aadA1, aph(3”)-lb, aph(6)-ld], sul2 and dfrA1 [41]. The risk factor analysis determined that the feeding of unpasteurised waste milk (“nonsaleable milk”) increased the odds of ESC-R *E. coli* being excreted by 1.61-fold (95% confidence interval (CI): 1.18; 2.18) as did the regular use of the third generation cephalosporin, ceftiofur, on farm (odds ratio (OR) 3.83, 95% CI: 2.29; 6.39) [20]. However, it is important to note the feeding of waste milk as a risk factor was only analysed as a “herd level factor”, rather than at the individual animal level. As such, any increased odds of ESC-R *E. coli* being shed on these farms may be due to a variety of management and environmental factors rather than being directly linked to waste milk feeding. Furthermore, the parenteral administration of antimicrobials to calves enrolled in this study was also permitted, which may have influenced the presence of AMR bacteria in faecal samples. A recent study by Pereira and colleagues from the USA determined that calves treated prophylactically with a single injection of enrofloxacin or tulathromycin to prevent bovine respiratory disease produced faecal samples with higher proportions of ciprofloxacin-resistant *E. coli* than untreated calves, even when all calves were fed waste milk [42].

Foutz and colleagues investigated the influence of a variety of waste milk and milk replacer feeding regimens on the presence of resistant *E. coli* in faecal samples from dairy calves on 15 farms in the USA [26]. Five newborn calves from each farm were assigned to receive either pasteurised nonsaleable milk (PNM) (containing multiple antimicrobial residues), commercially available medicated milk replacer (MMR) formulations (containing neoterramycin, neomycin, oxytetracycline and chlortetracycline) or non-medicated milk replacer (NMR). A total of 25 calves were included in each of the PNM and MMR groups, and 24 in the NMR group. Faecal and PNM samples were collected at weeks 1, 3, 5 and 16 of age. Antimicrobial susceptibility testing was carried out on three colonies of *E. coli*, which were randomly selected from each sample [26]. The antimicrobial resistance score was equally high in samples collected from calves receiving MMR or PNM at week 1 and 3 of age. At 5 weeks of age (before weaning), the resistance score was highest for calves fed MMR, followed by PNM, and then NMR. However, by week 16 (after weaning), there was no difference in the antimicrobial resistance levels in *E. coli* isolated from calves in the three feeding groups [26].

A risk assessment model (Monte Carlo simulation) to assess how waste milk fed to dairy calves influenced the transmission and faecal shedding of ESBL-producing *E. coli*, based on the results of previous studies was published by Awosile and Smith in 2017 [21]. The model focused on calves shedding ESC-R *E. coli* after being fed waste milk containing these resistant bacteria. The authors reported that the overall risk of shedding such resistant *E. coli* when calves were fed waste milk was around 57 calves per 10,000 head in the pre-weaning period. The model calculated that a 3% reduction in the prevalence of resistant *E. coli* in waste milk would contribute to a 50% reduction in the daily risk of excreting such pathogenic bacteria. Therefore, actions such as the pasteurisation of waste milk would be expected to reduce the overall level of risk to the calf and the wider environment. The study authors pointed out that in the risk assessment model neither the horizontal transfer of resistance genes nor the effect of antimicrobial residues on the selection for resistant bacteria was taken into account. However, it was assumed by the study authors that the antimicrobial residues contained in waste milk would also increase the risks of, e.g., the spread of antimicrobial resistant bacteria in the calf’s environment [21].

With respect to feeding colostrum containing antimicrobial residues, Tetens and colleagues from Germany analysed the risk of selecting for ESBL-producing *E. coli* when feeding this type of unsaleable milk [35]. Two farms with similar production systems and yields were selected. Both farms used the same intramammary antimicrobial dry cow preparations (a narrow-spectrum penicillin combination with an aminoglycoside). However, one farm followed a selective drying off system, where only cows with a SCC of more than 100,000/mL were treated with antimicrobial products, while the remaining cows received a non-antimicrobial teat sealer. By contrast, the other farm carried out blanket dry cow therapy, where all cows received antimicrobial dry cow products, regardless of udder health. All calves received only colostrum from their dam until the third day of age and did not receive antimicrobial treatment for disease. The authors were able to demonstrate a significant increase in the excretion of ESBL-producing *E. coli* on the third day post-partum when colostrum from cows from a herd using blanket dry cow therapy was fed to calves [35]. It is, however, important to note that these results were obtained by comparing two different commercial farms and other management factors, such as overall antimicrobial use, may have influenced the presence of AMR bacteria in this study. 

The results reported by Tetens and colleagues were in contrast to those studies reviewed in the EFSA Scientific Opinion, where it was described that feeding colostrum did not provide evidence of an increase in AMR bacteria, and that the colostrum does not contain measurable residues so long as the withdrawal period is observed and early calving does not occur [3,10]. In the German study, the dry period was sufficiently long, and penicillin was also largely used in combination with aminoglycosides as drying off agents, so that the conditions are largely comparable with those of Duse and colleagues [10,35]. The variation in these results could possibly be explained by the different sampling periods. Tetens and colleagues took samples on the third and 21st day post-partum and could only prove a significant increase on the first sampling date. On the 21st day, there was no longer any significant difference between drying off practices. In the study by Duse and colleagues, farmers themselves sampled the calves between the seventh and 28th day [10]. It would be interesting for further research to have closer sampling intervals, so that a decrease in prevalence could be assessed more precisely and, if necessary, more consideration of the initial situation on the farm could be given.

Most importantly, it is essential to emphasise that in the German study by Tetens and colleagues in the group with blanket dry cow therapy, six of 25 cows were heifers that did not receive any antimicrobial dry cow therapy (as they were non-lactating prior to calving); accordingly, their colostrum was antimicrobial-free. In contrast, in the group with selective dry cow therapy, nine out of 25 cows were treated with antimicrobials based on the predefined udder health criteria [35]. However, the researchers did not distinguish between calves that received untreated colostrum and those that received colostrum from antimicrobial-treated cows. It is not clear from the study results what influence this feeding difference might have had but is a limiting factor when considering the outcome of this study. It would certainly have been advantageous to include only antimicrobial-treated cows in a group that was by definition “all cows treated with antimicrobial DCT” and to have a control group with untreated cows as a comparison. Similarly, the fact that this study compared two different commercial farms with (presumably) differing antimicrobial use levels, treatment and management procedures may also have had an influence on the selective pressure for AMR bacteria in these calves. As such, given how essential colostrum feeding is for calf health, more research is needed into the effect of antimicrobial DCT on the presence of AMR bacteria in calves.

### 3.2. Composition of the Calves’ Microbiome

A study by Deng and colleagues in China investigated the influence of waste milk feeding on bacterial colonisation [23]. Rumen, caecum, colon and faeces were sampled and analysed with respect to feeding a diet of either pasteurised waste milk (PWM), acidified (otherwise untreated) waste milk (AWM), untreated waste milk (UWM) or bulk tank milk (UBM). It was found that the different milk diets had a great influence on bacterial richness and diversity [23]. The authors pointed out that feeding untreated waste milk is not suitable for calves. The reason for this is the increased occurrence of disease-associated bacteria in the microbiome in all areas of sampling. For example, in the AWM group, an increase in the abundance of pathogenic *Clostridia* spp. was noted, which is associated with intestinal inflammation and intestinal dysbiosis. Furthermore, when calves were fed untreated waste milk, increased gene expression associated with metabolic diseases was documented in the caecum and faeces [23]. 

Yousif and colleagues investigated the effects of feeding milk replacer both with and without antimicrobials added. The control was unmedicated, while the LCA (“low cocktail of antibiotics”) milk replacer had penicillin (0.024 mg/L), streptomycin (0.025 mg/L), tetracycline (0.1 mg/L) and ceftiofur (0.33 mg/L) added; ceftiofur (0.33 mg/L) was added to the LSA (“low concentration of a single antibiotic”) milk replacer [36]. The concentrations of antimicrobials added to the milk replacer were based on the authors’ analyses of waste milk on this farm; the median amount was then added to the milk replacer. A total of 12 male Holstein calves were included in the study, four calves in each feeding group. The calves were slaughtered at 35 days of age and samples from the ileum and colon, as well as faecal samples, were investigated by PCR and bacteriological culture. The LCA diet significantly reduced (*p* = 0.02) the level of Enterobacteriaceae (including *E. coli*) found in the intestines compared to the intestines of calves fed unmedicated milk replacer. The authors concluded that feeding a low cocktail of antimicrobials in milk replacer led to a shift in the bacterial taxa of intestinal microbiome, including a reduction in *E. coli*, which might have a positive effect on calf health by reducing the occurrence of diarrhoea in young calves [36].

A similar study by Li and colleagues (including Yousif and other co-authors from the previously mentioned study) also compared the microbiome under different feeding regimes [28]. Two groups of calves were compared, one control group fed untreated milk replacer and one group fed milk replacer with four antimicrobials added (namely penicillin (0.024 mg/L), streptomycin (0.025 mg/L), tetracycline (0.1 mg/L) and ceftiofur (0.33 mg/L)). In this case, however, antimicrobial residues were found to have only minor influences on bacterial diversity in the ruminal microbiota [28].

Maynou and colleagues in Spain analysed the effects of feed management on the diversity of bacterial flora in faeces and the upper respiratory tract [32]. Two groups of 20 calves each were fed either untreated milk replacer or pasteurised waste milk containing antimicrobial residues. The observation period was 49 days. The diversity of the bacteria remained relatively constant in both nasal and faecal samples. Only the composition of the nasal bacterial community differed, with calves fed untreated milk replacer having a significantly greater relative abundance of the Streptococcaceae family and the genus *Histophilus* (*p* < 0.05) in their nasal microbiota than those fed pasteurised waste milk. Within the intestine, a small difference could only be detected at a low taxonomic level. Overall, a small and non-specific influence of antimicrobial residues was documented [32].

An investigation into the functional profile of faecal bacteria up to six weeks of age in calves fed milk with or without antimicrobial residues was described by Pereira and colleagues at Cornell University [33]. A small group of 15 calves were fed raw milk with low concentrations of the antimicrobials, ceftiofur (0.1 µg/mL), penicillin (50 µg/mL), ampicillin (0.01 µg/mL) and oxytetracycline (3000 µg/mL) added, while the control group of 15 calves received untreated raw milk. The antimicrobial concentrations were based on previous research on waste milk residues by the authors [13,43]. Significant differences were determined between the two feeding groups with respect to the abundance of genes in faecal bacteria for stress response, nitrogen metabolism, regulation and cell signalling after one week of feeding, but not after three or six weeks of the study regimen [33]. 

Feng and colleagues investigated the effect of feeding milk containing the antimicrobial, pirlimycin (of the lincosamide class of antimicrobials), on the faecal microbiome of dairy calves [25]. Two groups of five calves were fed either pasteurised whole milk or pasteurised whole milk with the addition of pirlimycin (200 ng/mL). The dams of all calves enrolled in the study had received antimicrobial dry cow treatment with the first-generation cephalosporin, cephapirin benzathine. The amount of pirlimycin added was estimated based on the concentration present in milk collected at eight consecutive milkings from three healthy cows administered a licensed intramammary treatment containing this antimicrobial. Faecal samples were collected from all calves on day 1 (prior to feeding), day 42 and day 84 [25]. No significant differences were determined between feeding groups with respect to bacterial cell functions or the abundance of the major antimicrobial resistance types. The most abundant resistance genes were to tetracyclines; however, this was expected, as these resistance genes are commonly found in the environment and in animals never administered antimicrobials [25].

The aim of the study by Zhang and colleagues was to investigate the long-term effects of different feeding regimes on the development of the rumen and its bacterial composition. Whole milk, waste milk and milk replacer were compared in a total of 54 Holstein calves [37]. The experiment began at seven days of age and continued until the calves were 180 days old in order to understand the long-term effects of early feeding on the rumen. Three male calves in each treatment group were slaughtered at 58 days of age to allow for measurements of the gastrointestinal tract to be carried out. When waste milk was fed, a change in rumen bacterial structure was documented at two months of age [37]. At six months of age, however, this difference could no longer be detected. In terms of bacterial richness, the highest level of diversity was seen in the experimental group fed with waste milk. Furthermore, the calves in this group consumed less starter feed. This correlates with the significantly lower mass of the four stomachs as a proportion of calf liveweight at 58 days (*p* < 0.05) in the group fed waste milk, although the proportion of abomasum was significantly higher in this group. As the rumen flora is also significantly influenced by the starter culture, feeding waste milk, therefore, has indirect effects on the rumen microbiome [37].

Overall, the research described here is inconclusive with respect to the effect of waste milk feeding, leading to changes in the calves’ microbiome. Deng and colleagues described an increased occurrence of pathogenic bacteria through the feeding of waste milk, although the composition and distribution of bacterial genera are similar in all feeding regimes up to 21 days of age in that study [23]. In contrast, Yousif and colleagues reported a decrease in pathogenic bacteria after feeding milk replacer containing antimicrobials at residual levels, and Li and colleagues could not detect any difference in the composition of the microbiome on days 15, 25 and 35 of medicated milk replacer feeding [28,36]. Feng and colleagues could not determine any significant differences in bacterial functional profiles or resistance genes, and Maynou and colleagues found only minor differences [25,32]. Zhang and colleagues documented an increased bacterial diversity at two months of age when calves were fed with waste milk, but this diversity had waned by six months of age [37].

It is important to note that these studies differ with regard to feeding management, sampling frequency, detection method and bacteria examined, making direct comparisons challenging. While some of these studies used “natural” waste milk obtained from antimicrobially treated cows [23,32,37], others experimentally “spiked” milk or milk replacer with antimicrobials [25,28,33,36]. Both types of feed trial have merit: the artificially spiked milk obviously allows for a more exact comparison between feed groups, as the precise concentration of antimicrobials is known; however, spiked milk does not necessarily recreate real world conditions or allow for the possible pharmacokinetic changes, which may occur between treatment of the cow and the subsequent inclusion of antimicrobial residues in waste milk in the udder. The EFSA Scientific Opinion also addressed the direct effect on the calf’s bacterial flora [3]. Limiting the diversity of intestinal microbes is known to lead to poorer health [18,19], which also has far-reaching consequences for the development of the immune system [17]. However, the conclusions of those studies were not based on experiments on calves fed on waste milk. On the other hand, the feeding of waste milk has not been shown to lead to a deterioration in microbial diversity in the studies included here

### 3.3. Growth and Health Parameters

A further Chinese study by Zou and colleagues was concerned with the assessment of growth performance, serum metabolic profile, immunity and intestinal development in 84 Holstein calves, with respect to the feeding management of waste milk compared to bulk tank milk [38]. When milk composition was compared, there was no significant difference in milk protein percentages between milk types, although untreated waste milk did contain significantly more milk fat and solid non-fat as a percentage than bulk tank milk. A higher daily liveweight gain following the feeding of waste milk compared to bulk tank milk, and a positive influence on the serum profile were described [38]. An upregulation of immune components was reported for all forms (untreated, pasteurised and acidified) of waste milk feeding. In contrast, there was evidence that an inflammatory reaction in the jejunum and ileum occurs in both untreated and acidified waste milk. Overall, this study determined that bulk tank milk was a better source of feed for calf health compared to waste milk [38].

In the afovementioned study by Li and colleagues, the effects on calf growth and ruminant fermentation within the first 35 days were investigated [28]. Parameters such as body weight, height at withers, body length and heart circumference were used. Additionally, rumen fluid was analysed for pH, volatile fatty acids and ammonia concentration [28]. No differences between any of these parameters could be determined whether calves received milk replacer with, or without, antimicrobial residues. With respect to calf health, a comparatively lower frequency of diarrhoea at four weeks of age, longer ruminal papillae and an increased acetic acid concentration in the rumen were described in the group receiving milk replacer containing antimicrobial residues [28].

Zhang and colleagues reported a significantly lower average daily liveweight gain at 58 days (*p* < 0.05) among calves fed untreated milk replacer compared to those receiving waste milk [37]. However, the feed composition analysis showed that the milk replacer used in this study had a much lower crude protein (CP) and crude fat (CF) percentage than the waste milk fed to calves (CP 2.71 vs. 4.29%; CF 1.90 vs. 3.91%, respectively). Furthermore, this difference was no longer significant between treatment groups from 58–180 days of age.

In a recent study of Chilean dairy farms, Calderon-Amor and Gallo were able to demonstrate the effect of waste milk feeding on calf health based on the results from farm management interviews with 29 farm managers (covering approximately 700 calves) [22]. Health assessments of calves (e.g., body condition score, nasal discharge, the presence of diarrhoea, etc.) were carried out by one researcher. A milk composition analysis was not available. Just over half of the farms visited (51.7%) fed unpasteurised waste milk to their calves. Although the large confidence intervals mean that these results must be treated with caution, the authors calculated that calves fed with untreated waste milk had significantly higher odds of developing diarrhoea than calves fed treated (i.e., pasteurised or acidified) waste milk (OR 31.02; *p* < 0.05, 95% CI: 5.65; 170.21) [22].

In a small study of 18 healthy calves in Spain, Maynou and colleagues determined that calves fed pasteurised waste milk weighed 6.0 kg more at 42 days of age than calves in the control group fed untreated milk replacer (*p* < 0.05) [32] In this study, the authors attempted to make the diets nutritionally comparable, as such, the milk replacer was reported to contain 26% crude protein and 31% fat (based on dry matter (DM)), whereas the waste milk contained 28.4% crude protein and 30.1% fat (based on DM). The calves had equal access to water and the same calf starter diet. The differences between the waste milk and milk replacer groups with respect to average daily liveweight gain (kg/d), the liveweight gain to feed ratio as well as calf starter intake (kg) per day were also statistically significant (*p* < 0.05) [32].

Although not the primary focus of the study, an American group of researchers analysing the functional profile of faecal bacteria in calves fed raw milk containing antimicrobials found no significant differences between the calves fed drug residues and the control group (fed raw milk without residues) with respect to average liveweight gain [33].This study did not provide a milk composition analysis; however, it appears that the raw milk used for both groups came from the same source as stated in a previous paper [13].

In summary, Maynou and colleagues [32] were able to demonstrate a positive correlation between liveweight gain and feed intake with waste milk, as did Zou and colleagues [38]. Zhang and colleagues [37] reported a similar gain with experimentally spiked milk replacer. On the other hand, Li and colleagues [28] and Pereira and colleagues [33] did not determine a difference with regard to liveweight and two recent studies reported an increased frequency of diarrhoea when artificially spiked raw milk or milk replacer was fed [22,28].

In the EFSA Scientific Opinion, results on calf health and growth were similarly inconclusive [3]. No significant difference could be found with respect to liveweight gain when waste milk was fed in some studies [8,11,13,14]. By contrast, other groups have determined increased weight gain when waste milk is fed [12]. The studies of Langford and colleagues and Thames and colleagues did not find any increase in the occurrence of diarrhoea when feeding waste milk [3,11,14]. Brunton and colleagues even reported a decrease in the frequency of umbilical infections and diarrhoea [12]. However, it should be noted that these type of health and growth rate parameters are multifactorial, subject to the nutritional composition of the milk fed, as well as large individual variations, and often do not depend on feed management alone. 

### 3.4. Transmission of Pathogenic Bacteria

A study carried out by Edrington and colleagues in the USA looked at the effects of pasteurisation of waste milk on the presence of *Salmonella* spp. in calf faeces, as well as the pathogen’s serotype and sensitivity to antimicrobials [24]. Two groups of calves were fed with either pasteurised or untreated waste milk. Samples of waste milk were taken on six occasions and tested for the presence of *Salmonella* spp., *E. coli* and faecal coliforms. Weekly faeces samples were collected during the first to fourth week of age and at weaning at two months old. No significant differences were found between the feeding groups with respect to the prevalence of *Salmonella* spp. and antimicrobial susceptibility [24]. Only the serotypes differed. Contrary to expectations, no benefit from pasteurisation could be demonstrated. This is explained by the possible introduction of *Salmonella* from the environment; however, it is important to note that only one of the twelve waste milk samples tested contained *Salmonella* spp. A further influence would be possible from the infection of the calves shortly after birth, so that the *Salmonella* prevalence correlates more with the farm of origin than with the treatment of the waste milk. Consequently, the authors argue that feeding either pasteurised or untreated waste milk was not important in relation to *Salmonella* colonisation of calves [24]. Nevertheless, the advantages of pasteurisation outweigh the potential risks of untreated milk, so that a recommendation for treatment is given.

A further study on the presence of antimicrobial drug resistance and residues, including a survey of farm management practices with respect to calf feeding and antimicrobial use, was conducted by Tempini and colleagues on 25 dairy farms in the USA [34]. Waste milk tanks on the majority (72%) of these farms contained milk from treated cows as well as freshly calved animals, the remainder only contained milk from treated cows. The vast majority of farms (96%) in this study used antimicrobial DCT, with 87.5% using a blanket DCT to all cows in the herd. The most commonly used antimicrobial for DCT was the HPCIA ceftiofur in 40% of herds, followed by cephapirin (20%), penicillin (20%) and cloxacillin (8%). Ceftiofur was also the most common antimicrobial used to treat udder disease in lactating cows (68% of farms), respiratory disease (68%), reproductive disorders (76%) and lameness (40%) [34]. Out of a total of 25 waste milk samples from these farms (one raw sample from each farm) tested for the presence of antimicrobial drug residues, 60% were positive. Of these 25 samples, 11/25 (44%) contained beta-lactams (most frequently the HPCIA, ceftiofur), while 4/25 contained tetracyclines. The waste milk samples were also found to contain common mastitis pathogens, such as *Streptococcus* spp. (21/25; 84%), *Staphylococcus* spp. (20/25; 80%) and *E. coli* (10/25; 40%) [34]. Almost all farms (95%) in this study fed waste milk to their calves, and 20% of these farms did not pasteurise their waste milk prior to feeding. It was also reported that around two-thirds of farms fed milk replacer, either alone or mixed with waste milk. When milk replacer was used, around half of the farmers added either neomycin or chlortetracycline to it. Antimicrobial susceptibility testing was carried out on the *E. coli* isolated from waste milk samples. Multidrug resistance was determined in 20% (2/10) of isolates, while 40% (4/10) were susceptible to all antimicrobials tested [34]. The authors concluded that feeding calves waste milk containing residues of antimicrobials that are considered critically important to both human and veterinary medicine was a public health issue. The presence of mastitis pathogens in waste milk also demonstrated the importance of pasteurising waste milk before feeding to young calves [34].

In summary, the results presented by Edrington and colleagues do not show a significant difference in the excretion of *Salmonella* spp. when fed with pasteurised or untreated waste milk [24]. Tempini and colleagues demonstrated that a very high proportion (>80%) of waste milk samples contained mastitis pathogens, many of which were resistant to antimicrobials, some even multidrug resistant [34].

The studies included in the EFSA Scientific Opinion dealt with the transmission of bacteria through the bulk milk or the effectiveness of pasteurisation for risk reduction [8,44]. The studies of Aust and colleagues and Fitzgerald and colleagues confirmed a positive effect of pasteurisation in terms of minimising the transmission of bacteria [8,44]. Aust and colleagues did not investigate the direct excretion prevalence but compared the bacterial concentration of pasteurised and untreated raw milk. Although an effective reduction in the bacteria could be demonstrated for both pasteurisation methods (72 °C for 15 s; 64 °C for 35 min), no complete inactivation by pasteurisation could be proven overall [8]. This was attributed to the prevailing hygiene standards, the different bacterial load of the waste milk, as well as possible recontamination after treatment. Nevertheless, Aust and colleagues noted the known reduction in the risk of transmission of *E. coli, Streptococcus agalactiae, Mycoplasma spp.* and *Mycobacterium paratuberculosis* after pasteurisation and the importance of the procedure [8].

## 4. Materials and Methods

The EFSA Scientific Opinion published in 2017 [3] serves as a basis to this narrative review. The EFSA report is a risk assessment for the possible development of antimicrobial resistance due to the feeding of waste milk to calves. However, as only studies already published up to the end of 2016 could be included in the EFSA report, an updated literature search was carried out here to reflect the current level of knowledge. This included a review of existing literature on the subject of antimicrobial residues in waste milk and their effects, particularly on commensal and pathogenic bacteria, when fed to calves. 

The literature was searched, evaluated and sorted according to relevance. The following databanks were searched: US National Library of Medicine/National Institutes of Health (PubMed), Scopus and Web of Science. Initially, “waste milk” or “discard milk” were used as “title/abstract/keywords”. The search was carried out for publications made available between January 2016 and December 2020. Additional keywords included “calf”, “antimicrobial” and “antibiotic” (as well as combinations of these as described in the Results section). 

## 5. Conclusions

The studies reviewed here obtained a wide variety of outcomes. While feeding waste milk containing antimicrobial residues appears to increase the excretion of antimicrobial-resistant bacteria in dairy calves, such shedding is frequently short-lived and transient. Although shifts in the calves’ microbiome were commonly reported following waste milk feeding, it is not possible at present to confirm whether these changes in diversity have positive or negative effects on calf health. Similarly, the transmission of antimicrobial resistant bacteria from waste milk to calves appears to be an extremely complex phenomenon. It is important to note that the majority of the studies reviewed here only examined the resistance pattern of *E. coli*. Although *E. coli* is one of the most common bacteria found on dairy farms, these results should be verified in the future by investigating other bacterial pathogens and other commensal bacteria, such as *Enterococcus* spp. The partially divergent results of the studies included here can be explained by a variety of factors, such as the frequency of sampling, the overall sampling period, the selection and number of antimicrobial classes tested for bacterial resistance and also the bacterial species tested. In addition, the composition and treatment of the waste milk, with respect to different antimicrobial classes and concentrations, is an additional complicating factor.

## Figures and Tables

**Table 1:** Summary of studies published between 2016–2020 and included in this narrative review.

Author/Year	Sample Size Calculation	Control Group	Experimental /Commercial Farm	Study Type	Randomisation? Convenience Sample?	Parenteral Use of Antibiotics Permitted in Study Animals?
**Awosile et al. (2018)** [20]	No	No	Commercial	Screening	Convenience sample of 8 farms—no detail on animal selection	Yes
**Awosile and Smith (2017)** [21]	N/A	N/A	N/A	Risk assessment model	N/A	N/A
**Calderon-Amor and Gallo (2020)** [22]	No	No	Commercial	Observational survey	Convenience sample	Yes
**Deng et al. (2017)** [23]	No	Yes (bulk milk)	Commercial	Inventional	1 farm—calves randomly assigned to treatment groups	Not mentioned
**Edrington et al. (2018)** [24]	No	No (both groups fed WM)	Commercial	Longitudinal	4 farms—groups not randomised, more animals fed pasteurised WM due to farmer concerns on feeding non-pasteurised WM	Not mentioned
**Feng et al. (2020)** [25]	No	Yes (pasteurised whole milk)	Experimental	Feeding trial/intervention	1 farm—10 calves selected at birth	Only vaccinations and vitamins mentioned
**Foutz et al. (2018)** [26]	No	Yes (non-medicated milk replacer)	Commercial	Prospective cohort	Convenience sample	Yes, for both clinical disease and prophylaxis
**Horton et al. (2016)** [27]	Yes	No (all calves fed WM)	Commercial	Longitudinal	1 farm—no details on animal selection	Not mentioned
**Li et al. (2019)** [28]	Yes	Yes (non-medicated milk replacer)	Experimental	Intervention/feed trial	1 farm—randomised block design to account for birth date	Not mentioned
**Manga et al. (2019)** [29]	No	No (but not clear which calves fed WM)	Commercial	Observational/monitoring	1 farm—no details on animal selection	No
**Maynou et al. (2017a)** [30]	No	Yes (non-medicated milk replacer)	Commercial	Intervention/feed trial	8 farms—selected according to herd size, calf feeding management	No—calves excluded if treated with AB before day 42
**Maynou et al. (2017b)** [31]	No	Yes (non-medicated milk replacer)	Experimental	Longitudinal	Sourced from 3 commercial farms—no details on animal selection	No—calves excluded if treated with AB at any time
**Maynou et al. (2019)** [32]	No	Yes (non-medicated milk replacer)	Experimental	Intervention/feed trial	Sourced from 3 commercial farms—calves assigned to treatment by farm of origin and body weight	No—calves excluded if treated with AB before day 42
**Pereira et al. (2018)** [33]	No	Yes (with raw milk)	Experimental	Controlled feeding trials	Randomized (calves from a local dairy farm)	Not mentioned
**Tempini et al. (2018)** [34]	Yes	No	Commercial	Cross-sectional	Convenience samples of 25 farms	-
**Tetens et al. (2019)** [35]	No	No	Commercial	Comparative cohort	Convenience sample: 2 farms chosen because of similar herd size and milk production	Yes (β-lactam treatments prohibited)
**Yousif et al. (2018)** [36]	No	Yes (non-medicated milk replacer)	Experimental	Feed trial	12 calves randomly assigned to feed groups	No
**Zhang et al. (2019)** [37]	No	Yes (raw milk)	Experimental	Longitudinal	1 farm—no details on animal selection	Not mentioned
**Zou et al. (2017)** [38]	No	Yes (bulk milk)	Experimental	Interventional	1 farm—similar birth weight	Yes, calves with severe diarrhoea received injections of gentamycin sulphate

All studies were non-blinded. WM = waste milk, AB = antibiotics.

## Supplementary Materials

The following are available online at https://www.mdpi.com/2076-0817/10/2/112/s1, Table S1: Summary of studies published between 2016–2020 and included in this narrative review–demographics, results and study descriptions, Table S2: Aspects considered in the studies dealing with feeding waste milk and published since 2016.
