# Supplementary material for: The Effects of Feeding Waste Milk Containing Antimicrobial Residues on Dairy Calf Health
Source: Pathogens. 2021 Jan 22;10(2):112. doi: 10.3390/pathogens10020112 (PMC7911522; doi:10.3390/pathogens10020112)
Supplement: Supplementary file 1 [file pathogens-10-00112-s001.pdf]

Table S1: Summary of studies published between 2016-2020 and included in this narrative review – demographics, results and study descriptions

**Author & Year [Ref.]**	**Country**	**Number of animals**	**Effects of waste milk feeding**	**Feeding groups**	**Antimicrobials assessed in feed**	**Sampling**	**Waste milk - natural or artificially spiked with antimicrobials**
**Awosile et al, (2018) [20]**	Canada	488 calves	*Salmonella enterica* isolated in 3.3% of samples, all were susceptible to all antimicrobials tested.<br>Faecal carriage of *E.coli* with reduced susceptibility to cephalosporins (ESC) reported at frequency of 81.2% using selective culture. All tested isolates were multi-drug resistant. Feeding of unpasteurised nonsaleable milk associated with faecal recovery of ESC.	Unpasteurised waste milk was fed on these farms	Ceftiofur most commonly used (but not assessed here)	Faecal samples from calves<br>1) Between day 2-15<br>2) Between day 42 and 56	Natural
**Calderon-Amor & Gallo (2020) [22]**	Chile	700 calves	Factors associated with diarrhoea included using milk replacer or untreated waste milk compared to pasteurised/acidified waste milk	Waste milk was fed on these farms<br>-	(Survey)	Survey of management. Calf health assessed at one on farm visit	Natural

Table S1: Summary of studies published between 2016-2020 and included in this narrative review – demographics, results and study descriptions

**Deng et al, (2017) [23]**	China	84 calves	Differences in microbial richness (taxonomic groups) of bacteria; Increased prevalence of pathogenic bacteria; expression of genes related to metabolic diseases;	1) untreated bulk milk (UBM) 2) acidified waste milk (AWM) 3) pasteurised waste milk (PMW) 4) untreated waste milk (UWM)	Various (mixture) including ß-Lactams, Aminoglycoside, Tetracycline, Macrolide	12 calves randomly euthanised on day 21: Mucosa and digesta samples (rumen, caecum, colon, faeces), 3 animals per feeding group	Natural
**Edrington et al, (2018) [24]**	USA	211 calves	No statistically significant difference with respect to the excretion (yes/no) of *Salmonella* spp.; differences with the serotypes Majority susceptible to all antimicrobials tested	1) Pasteurised waste milk (PWM); 128 calves; 2) Nonpasteurised waste milk (NPWM); 83 calves	Not known	WM & faecal samples: weekly at 1-4 weeks of age, & at weaning.	Natural
**Feng et al, (2020) [25]**	USA	10 calves	No effect on bacterial cell function categories -except in the “phage, prophage and transposable elements” category, which indicates increased susceptibility of the calf microbiome to the transfer of antimicrobial resistance;	1) Pasteurised whole milk 2) Pasteurised whole milk with pirlimycin added	Pirlimycin (0.2mg/L) (day 2 to 50)	Faecal Samples on day 1, 42 and 84 before feeding	Artificial

Table S1: Summary of studies published between 2016-2020 and included in this narrative review – demographics, results and study descriptions

**Foutz et al. (2018) [26]**	USA	74 calves	Calves fed MMR and PNM more frequently excreted multi-resistant bacteria, overall resistance levels fell over time, no difference between feeds at 16 weeks.	1) PNM (pasteurised nonsaleable milk) 2) MMR (medicated milk replacer) 3) NMR (nonmedicated milk replacer)	MMR contained: neoterramycin, neomycin, oxytetracycline, chlortetracycline. PNM contained ampicillin, cephapirin, penicillin, ceftiofur	Faecal samples (week 1, 3, 5, 16)	Natural/Medicated MR
**Horton et al, (2016) [27]**	UK	250 Cows and 40 unweaned Calves	93 % of the faecal samples (calves, cows) contained *E.coli* isolates with CTX-M genes, 100% of the unweaned calves carried CTX-M *E.coli*, prevalence of CTX-M *E.coli* decreased with age	Waste milk was fed on this farm	Most frequently detected were cefquinome and cefalexin in waste milk	Faecal samples, waste milk samples, environmental samples; six visits with longitudinal sampling of calves (n=20), follow up 1 year later	natural
**Li et al, (2019) [28]**	China	20 bull calves	No difference seen in the diversity of the microbiome, no effect on liveweight. lower incidence of diarrhoea in spiked milk replacer group. Stimulated development of ruminal papillae.	1) Milk replacer spiked with antibiotics (ANT) 2) Milk replacer without antibiotics (CON)	Penicillin (0.024mg/l), Streptomycin (0.025mg/l), Tetracycline (0.1mg/l), Ceftiofur (0.33 mg/l)	Rumen fluid: 15., 25., 35. Tag for pH, volatile fatty acids and NH_3-N; 10 samples from day 35 investigated for microbial community; Faecal consistency and feed intake daily; Liveweight, withers height,	Artificial

Table S1: Summary of studies published between 2016-2020 and included in this narrative review – demographics, results and study descriptions

						body length and heart girth: Day 1, 7, 14, 21, 28, 35	
**Manga et al, (2019) [29]**	Czech Republic	13 calves	94 % of the faecal samples contained cefotaxime-resistant E.coli (CREC) using selective culture, later confirmed as AmpC-producing; all calves were positive on day 1 or 2, 90 % of cefotaxime-resistant *E.coli* were multi-resistant; Frequency of isolation of CREC decreased with age	Calves were fed milk with antimicrobial residues on this farm – no further details	AM Usage on the farm included lincomycin, oxytetracycline, amoxicillin/ clavulanic acid, marbofloxacin, cefquinome, cefoperazone	Faecal samples from calves until day 63, environmental samples, feed samples, faecal samples from the 13 dams before calving	Natural
**Maynou et al (2017a) [30]**	Spain	20±5 calves	Increased excretion of resistant and multi-resistant *E. coli* (9 antimicrobials tested),<br>reduced excretion with calf age.<br>Increased prevalence of colistin-resistant *Pasteurella multocida;*<br>Calf isolates similar to environmental ones, not to those from the dams	1) Waste milk (WM)<br>2) Milk replacer (MR)	Not known	Rectal swabs (for *E.coli*) und nasal swabs (for *Pasteurella multocida*):<br>42±3.2 days., 1 year;<br>environmental samples	Natural

Table S1: Summary of studies published between 2016-2020 and included in this narrative review – demographics, results and study descriptions

**Maynou et al, (2017b) [31]**	USA	52 female calves (3 ± 1.3 days old)	Increased excretion of resistant *E. coli* (12 antimicrobials tested), reduced excretion with calf age.	1) Pasteurised waste milk (pWM) 2) Milk replacer (MR)	Not known	Rectal swabs: Study days 0, 35, 56	Natural
**Maynou et al, (2019) [32]**	USA	40 calves (2-5 days old)	Non-specific influence on gut microbiota, Increased liveweight gain	1) Pasteurised waste milk (pWM) 2) Milk replacer (MR)	Beta-lactam antimicrobials were detected in each batch of waste milk, other antimicrobials might also be present.	Faecal samples, nasal swabs, liveweight: twice a week. Microbiome analysed: Day 42	Natural
**Pereira et al, (2018) [33]**	USA	30 calves	Significant difference in relative abundance of functional genes in bacteria, particularly those expressing for stress responses, regulation and cell signalling, and N metabolism.	1) Raw milk with antimicrobials added (DR) 2) Raw milk (NR)	Ampicillin, ceftiofur, penicillin, oxytetracycline	Faecal samples: Weekly until 6 weeks old	Artificial

Table S1: Summary of studies published between 2016-2020 and included in this narrative review – demographics, results and study descriptions

**Tempini et al, (2018) [34]**	USA	25 farms	60% of waste milk tested contained detectable concentration of at least 1 antimicrobial, 20% of *E.coli* (n=10) from waste milk were multidrug resistant	(Survey)	-	Milk samples from each farm Bulk tank / waste milk (1 sample/ farm)	Natural
**Tetens et al, (2019) [35]**	Germany	50 calves	Higher prevalence of ESBL producing *E.coli* on the farm using blanket dry cow therapy	1) Colostrum from farms with selective drying off 2) Colostrum from farms with blanket antibiotic dry cow therapy	Combination product (dihydro-streptomycin, nafcillin and penicillin-G procaine) for blanket DCT; Framecytin with penicillin or cloxacillin for selective DCT	Faecal samples: Day 3, 21 post-partum	Natural (colostrum)
**Yousif et al, (2018) [36]**	China	12 calves	Cocktail of antibiotics induced changes at different taxonomic levels, a decrease in *E.coli* was found in the group receiving the cocktail of antibiotics, which might reduce diarrhoea	1)Milk replacer without AB (CON) 2) Milk replacer with low cocktail of AB (LCA) 3) Milk replacer with one AB	LCA: Penicillin, streptomycin, tetracycline, ceftiofur, LSA: Ceftiofur	Calves euthanised on Day 35: Digesta from ileum and colon, faecal samples, IgG from blood samples	Artificial

Table S1: Summary of studies published between 2016-2020 and included in this narrative review – demographics, results and study descriptions

				(LSA)			
**Zhang et al, (2019) [37]**	China	54 calves (from 7 days of age)	Increased average daily weight gain, different bacterial community with WM	1) Whole milk<br>2) Pasteurised waste milk<br>3) Milk replacer	Gentamicin (only residue detected in WM)	Stomach mass: Day 58 (n=3, male calves).<br>Feed intake & growth:<br>Day 7, 14, 28, 49, 58, 90, 120, 150, 180 (female calves);<br>Blood samples, rumen fermentation:<br>Day 60, 180 (female calves)	Natural
**Zou et al, (2017) [38]**	China	84 male calves	Increased liveweight gain, increased diarrhoea incidence, increased inflammation of jejunum and ileum, no difference in immune response	1) Bulk tank milk (UBM)<br>2) Untreated waste milk (UWM)<br>3) Pasteurised waste milk (PWM)<br>4) Acidified waste milk (AWM)	Not known	Weight, Blood samples: Day 1, 22.<br>Diarrhoea: assessed daily<br>Small intestine tissue at d 22 (n=6)	Natural

Table S1: Summary of studies published between 2016-2020 and included in this narrative review – demographics, results and study descriptions

Table S2: Aspects considered in the studies dealing with feeding waste milk and published since 2016.

Author & Year	Excretion of AMR bacteria	Age of calves	Microbiome	Health	Transfer of bacteria
**Awosile et al (2018)** [20]	•	•			
**Awosile & Smith (2017)** [21]	•				
**Calderon-Amor & Gallo (2020)** [22]				•	
**Deng et al (2017)** [23]			•	○	
**Edrington et al (2018)** [24]	○				•
**Feng et al (2020)** [25]			•		
**Foutz et al (2018)** [26]	•	•			
**Horton et al (2016)** [27]	•	•			○
**Li et al (2019)** [28]			○	•	
**Manga et al (2019)** [29]	•				
**Maynou et al (2017a)** [30]	•	•			
**Maynou et al (2017b)** [31]	•	•			
**Maynou et al (2019)** [32]			•	○	
**Pereira et al (2018)** [33]	○	○	•	○	
**Tempini et al (2018)** [34]					•
**Tetens et al (2019)** [35]	•	•			
**Yousif et al (2018)** [36]			•		
**Zhang et al (2019)** [37]		•	•	○	
**Zou et al (2017)** [38]				•	

• = Primary focus of the study; ○ = Secondary focus of the study.
